# Environmental Metabolomics Promises and Achievements in the Field of Aquatic Ecotoxicology: Viewed through the Pharmaceutical Lens

**DOI:** 10.3390/metabo12020186

**Published:** 2022-02-17

**Authors:** Thibaut Dumas, Frédérique Courant, Hélène Fenet, Elena Gomez

**Affiliations:** HydroSciences Montpellier, IRD, CNRS, University of Montpellier, Montpellier, France; thibaut.dumas@umontpellier.fr (T.D.); helene.fenet@umontpellier.fr (H.F.); maria-elena.gomez-hernandez@umontpellier.fr (E.G.)

**Keywords:** metabolomics, pharmaceutical active compounds, aquatic organisms, mechanism of action, biomarkers, multi-omics, hazard assessment, biomonitoring

## Abstract

Scientists often set ambitious targets using environmental metabolomics to address challenging ecotoxicological issues. This promising approach has a high potential to elucidate the mechanisms of action (MeOAs) of contaminants (in hazard assessments) and to develop biomarkers (in environmental biomonitoring). However, metabolomics fingerprints often involve a complex mixture of molecular effects that are hard to link to a specific MeOA (if detected in the analytical conditions used). Given these promises and limitations, here we propose an updated review on the achievements of this approach. Metabolomics-based studies conducted on the effects of pharmaceutical active compounds in aquatic organisms provide a relevant means to review the achievements of this approach, as prior knowledge about the MeOA of these molecules could help overcome some shortcomings. This review highlighted that current metabolomics advances have enabled more accurate MeOA assessment, especially when combined with other omics approaches. The combination of metabolomics with other measured biological endpoints has also turned out to be an efficient way to link molecular effects to (sub)-individual adverse outcomes, thereby paving the way to the construction of adverse outcome pathways (AOPs). Here, we also discuss the importance of determining MeOA as a key strategy in the identification of MeOA-specific biomarkers for biomonitoring. We have put forward some recommendations to take full advantage of environmental metabolomics and thus help fulfil these promises.

## 1. Introduction

Since the early 21st century, scientists often set ambitious targets using environmental metabolomics to address challenging ecotoxicological issues. Numerous reviews on environmental metabolomics have underlined the high potential of this approach: (i) to gain insight into the specific mechanisms of action (MeOAs) through which contaminants achieve adverse outcomes at higher biological organization levels (e.g., cellular, organ, and individual) in comprehensive hazard or risk assessments, and (ii) to develop robust metabolic biomarkers of effects or exposure as an environmental biomonitoring tool [1,2,3,4,5,6,7,8]. This potential is based on the fact that metabolomics has the advantage of providing a high sensitivity (i.e., low levels of metabolites can be measured), high assessment efficiency (i.e., a broad range of metabolites may be measured without a priori), and comprehensive evaluation (i.e., provides an overview of molecular effects, reflecting the physiological status of the organism) [4,5]. The fulfilment of this high potential of metabolomics could, however, be hampered by its current shortcomings. Metabolomics outcomes (i.e., metabolic fingerprints) reflect a “simplified” picture of an extremely complex biological system. When differential metabolomics analyses are applied to measure changes in metabolite abundance in organism responses to contaminant exposure, the observed changes correspond to the overall molecular effect, which is not only directly related to the MeOA of the contaminants, but also to all processes that are geared towards maintaining cellular homeostasis under stress conditions [9]. Distinguishing the changes directly related to the MeOA from the overall molecular effect can therefore be a difficult task, especially when no prior knowledge on the contaminant MeOA is available. Moreover, MeOA related molecular changes may not be accessible for analysis, as metabolite detection is dependent on the analytical conditions, which means that only a fraction of the metabolome is often analysed. Similarly, identifying a set of metabolites specific to the response of organisms to contaminant exposure (i.e., biomarkers) is also a quite complicated task, as many changes in metabolite abundance may be caused by homeostasis processes that could simply be a common response to different stressors, and thus not contaminant-specific. Given the promising potential of environmental metabolomics and the current limitations, here we propose an updated review on the achievements of this approach in relation to ecotoxicological issues.

Pharmaceutical active compounds (PhACs) are known to be ubiquitous contaminants in aquatic environments [10,11,12], originally designed specifically to maximise their biological activity at low doses and to target certain metabolic, enzymatic, or cell-signalling mechanisms in target organisms (e.g., humans and livestock) [13]. Interestingly, MeOAs of PhACs are relatively well-known in target organisms. Such knowledge can therefore initially be used to investigate the MeOA of PhACs in aquatic organisms (non-target organisms), as molecular targets and/or pathways of toxicity can be conserved across species. Environmental metabolomics is currently becoming an essential approach to gain further insight into the molecular mechanisms involved in sublethal adverse outcomes of PhACs at low concentrations in aquatic organisms [14].

Hence, we decided to review environmental metabolomics achievements with regard to PhACs, as prior knowledge about their MeOA could help overcome some limitations of this approach. The understanding of MeOAs through environmental metabolomics (and multi-omics) will be discussed as a key strategy in the construction of adverse outcome pathways (AOPs) [15] for hazard assessment and in the identification of biomarkers for biomonitoring. This review also offers some recommendations with regard to the current literature, so as to help researchers take full advantage of this approach.

## 2. Literature Review Methodology

Published articles were screened from search engines such as Web of Science and Google Scholar. Different sets of keywords were used to identify published studies related to the investigation of PhAC effects on aquatic organisms throughout the metabolomics approach. These included “metabolomics”, “pharmaceuticals”, “ecotoxicology”, and “aquatic organism”. This literature review highlighted 30 articles published within the last decade. Among them, we decided to focus on articles that met the following selection criteria: (i) the exposure concentration was close to the environmental conditions (≤100 µg/L); (ii) measured metabolites in aquatic organisms were significantly modulated by the exposure (*p* < 0.05 and modulation amplitude > analytical variability, i.e., 30%) [16]; and (iii) metabolite identification was confirmed by the corresponding analytical standard injected in the same conditions and into the same analytical instruments as those used for the samples (confidence level 1) [17], or putative annotation was only accepted if supported by MS/MS spectra. The 100 µg/L threshold was chosen as the upper environmental concentration, likely to occur in areas of the world where wastewater is untreated, whereas the most common concentrations in fresh or marine waters tend to be less than 1 µg/L [18,19]. Finally, 16 articles published between 2014 and 2022 were selected [20,21,22,23,24,25,26,27,28,29,30,31,32,33,34,35]. Substantial information was extracted from each of them and is summarized in Table S1, such as the studied PhAC and its therapeutic class, the exposure conditions (time and concentrations), the studied organism and its sex, the sample type (e.g., organ, whole organism, and biofluide), and the analytical method used (e.g., mass spectrometry combined with liquid or gas chromatography (LC/GC-MS), and protonic nuclear magnetic resonance spectroscopy (^1^H NMR)), and significantly modulated metabolites and their associated metabolic pathways. When specified in the article, we have also provided information on (i) the physiological functions or biological processes concerned with the modulated metabolites; (ii) the supported MeOA of the PhAC; and (iii) the links of metabolomics data to other measured physiological, morphological, or biochemical endpoints. Each row of Table S1 corresponds to an exposure condition (i.e., a single concentration at a single exposure time). Thus, 44 experiments from the 16 selected studies are listed in Table S1.

## 3. General Information on the Corpus of Articles

**Figure 1:**
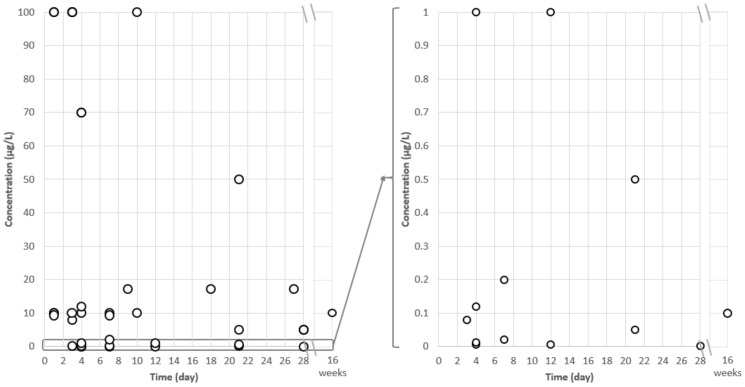
Relationship between PhAC exposure time (days) and concentration (µg/L) based on the findings of 44 experiments performed in the 16 studies selected in this review.

Among the 16 selected publications, fish and molluscs were the most studied aquatic organisms, including five fish species (*Danio rerio*, *n* = 4 articles; *Carassius auratus*, *n* = 1; *Oryzias latipes*, *n* = 1; *Oryzias melastigma*, *n* = 1; and *Pimephales promelas*, *n* = 1), three bivalve mollusc species (*Mytilus galloprovincialis*, *n* = 4; *Lampsilis fasciola*, *n* = 1; and *Saccostrea glomerata*, *n* = 1), one gastropod mollusc species (*Lymnaea stagnalis*, *n* = 1) and one crustacean species (*Hyalella azteca*, *n* = 1). The effects of 15 PhACs belonging to nine different therapeutic classes were investigated on these aquatic organisms, with antibiotics being the most studied therapeutic class (Table S1). Organisms were exposed to concentrations in the 0.001–100 µg/L range, mainly over periods ranging from 24 h to 28 days, with a maximum exposure period of 16 weeks (Figure 1). We noted an exposure time of less than 7 days in two-thirds of the experiments (*n* = 24). Similarly, there was an exposure concentration of >1 µg/L in two-thirds of the experiments (*n* = 27). The lowest exposure concentrations (≤1 µg/L) were quite well distributed between short (≤7 days) and (semi-)long term experiments (>7 days). However, we observed a lack of data on long-term exposure for the most frequent very low environmental concentrations.

Recent studies have documented serious PhAC-induced changes mainly in the metabolome of fish and molluscs, i.e., the most commonly studied organisms (Table S1). The reported changes in metabolite abundance after PhAC exposure were essentially related to the primary metabolism. As reported in Table S1, amino acid metabolism (e.g., phenylalanine, tyrosine, proline, and threonine), nucleotide metabolism (e.g., purine and pyrimidine), carbohydrate metabolism (e.g., glucose and maltotriose), lipid metabolism (e.g., fatty acids, glycerophospholipids, and glycerophosphocholines), and the citric acid cycle (e.g., fumarate, malate, and citrate) were the most impacted metabolic pathways in both fish and molluscs. The contrast between the findings on primary and secondary metabolites was not due to a lack of change in the secondary metabolism, but rather to a lack of knowledge regarding this metabolism in most aquatic species. Researchers have sought to clarify the biological meaning underlying these changes and their consequences by linking these impacted metabolites and metabolic pathways to different physiological and biological processes that could be disturbed. Regardless of the studied organism, these processes were mainly related to the energy metabolism, reproductive function, immune system, osmoregulation, protein turnover, or neuronal processes.

## 4. Application of Environmental Metabolomics to Address Ecotoxicological Issues: Case Studies on PhACs

This section summarises applications of environmental metabolomics when investigating the effects of PhAC exposure, and then demonstrates its potential to address several ecotoxicological issues. Environmental metabolomics is first applied to obtain an overview of the molecular effects on metabolome triggered by the exposure of aquatic species to PhACs. The experimental design of these studies was generally focused on the effects of a single PhAC (or more rarely its degradation product) in a time- and/or concentration-dependent manner (Table S1). For example, Cappello et al., demonstrated the effects of drospirenone (a synthetic progestin) on mussels (*M. galloprovincialis*) exposed for 7 days at four different concentrations, ranging from low environmental concentrations (20 and 200 ng/L) to higher concentrations (2 and 10 µg/L) [20]. Their metabolomics results revealed an effect of drospirenone on energy metabolism (down-modulation of glucose) in mussels exposed at a 20 ng/L concentration, while the effects tended to extend to other metabolic pathways (e.g., energy, amino acid, and glycerophospholipid metabolism) with increasing concentrations. They therefore concluded on the potential deleterious impacts of drospirenone on organisms, although no endocrine action was observed, as expected by the authors. Hence, environmental metabolomics studies succeeded in discriminating the response of organisms exposed to different low and high PhAC concentrations.

Ecotoxicology research is confronted with the issue of contaminant mixtures in the environment. Environmental metabolomics studies have also been carried out to address this issue by investigating the effects of PhAC mixtures on aquatic organisms [22,25]. De Sotto et al., studied the effects of the antibiotics florfenicol, clarithromycin, and sulfamethazine at a 100 µg/L concentration—individually and in mixtures—on *Danio rerio* fish exposed for 72 h [22]. Metabolome changes noted after individual exposure to florfenicol and clarithromycin were more numerous than for exposure to sulfamethazine. The main affected metabolic pathway was related to purine metabolism, especially guanosine involved in protected neurons against excitotoxic damage. The similarity between the florfenicol and clarithromycin findings might be related to their similar MeOAs, which inhibit protein biosynthesis interacting with the 50 S subunit of the organism. Unexpectedly, a few metabolites were modulated when the antibiotic mixture was tested, probably due to antagonistic interactions, although the authors provided no explanations for such interactions. Environmental metabolomics is therefore able to highlight different responses when organisms are exposed to compound mixtures compared to PhACs alone, while also revealing the type of interaction (i.e., additive, antagonistic, and synergistic). However, further investigations are needed to understand the mechanisms underlying contaminant interactions.

The environmental physicochemical features can differ from one region to another, and may also be modified by human activities and climate change. Ecotoxicology research has since integrated these environmental parameters in studies on organism responses to contaminant exposure. Environmental metabolomics was applied to study the effects of PhACs combined with different environmental parameters. For example, Mishra et al., assessed the pH-dependent toxicity of fluoxetine in embryonic zebrafish after 96 h post-fertilisation exposure [28]. Fluoxetine exposure (56 and 70 μg/L) caused no significant changes at pH 7, yet metabolic alterations were observed at pH 8 and 9 (70 μg/L fluoxetine). At pH 8, the impacted metabolites were D-glucose, D-glucose-6-phosphate, glycine, and urea, and at pH 9, changes were observed in 6-phosphogluconic acid, D-glucose-6-phosphate, lactic acid, lysine, glycine, and urea. Such fluoxetine-induced changes under higher pH conditions revealed the disruption of nitrogen waste excretion (urea accumulation), which could be toxic to embryos, as well as a consumption of reserve energy to supplement energy demand, thereby reflecting organism stress. The findings of this study were in line with the fact that fluoxetine is a basic ionogenic drug whose toxicity increases with increasing aqueous pH due to the greater bioavailability of its neutral form at a higher pH, and in turn to a greater rate of organism uptake of this compound from aqueous media [28].

Environmental metabolomics studies have thus demonstrated the ability of this approach to address a wide range of ecotoxicological issues related to the molecular effects of PhACs on aquatic species. This includes low concentration effects on non-target organisms, mixture effects, and combinations of PhACs with other environmental stressors. While environmental metabolomics is therefore able to generate evidence on the molecular effects of contaminants in close relation to the organism phenotype, there is also a need for the generation of more information about their MeOA in non-target organisms and how they can have adverse effects at low concentrations.

## 5. Environmental Metabolomics to Decipher Mechanisms of Action in Aquatic Species

In addition to providing an overview of the molecular effects triggered by contaminant exposure, environmental metabolomics has a high potential to decipher the contaminant MeOA via the identification of toxicity pathways and/or signatures [36]. Regarding examples dealing with PhACs, Fu et al., highlighted the MeOA of diclofenac in the *Hyalella azteca* crustacean exposed for 10 days to 10 and 100 µg/L concentrations [24]. Significant changes in metabolite abundance—more pronounced with increasing diclofenac concentrations—were measured, e.g., prostaglandin E1 (down-modulation), arachidonic acid (up-modulation), and three other prostaglandin metabolites. The authors therefore argued that diclofenac inhibits the activity of cyclooxygenases, which convert arachidonic acid to prostaglandin, in line with the known MeOA of diclofenac in humans and other aquatic organisms [37,38]. They also revealed a further pathway altered by diclofenac, i.e., the carnitine shuttle pathway, which could be investigated as another MeOA of diclofenac in *H. azteca*.

However, it can be a difficult task to gain insight into contaminant MeOAs when focusing solely on one molecular level (i.e., metabolite). Hence, the combined use of metabolomics and other omics approaches (i.e., genomics, transcriptomics, and proteomics) is a key strategy for in-depth exploration of the MeOA of PhACs and other contaminants [39]. Studies already carried out using this strategy have demonstrated its high potential in this field [23,32,34]. In a previous study, we used both metabolomics and proteogenomics approaches to assess *M. galloprovincialis* digestive gland samples, and obtained very complementary information on the effects of carbamazepine (3-day exposure; 0.08 and 8 µg/L concentrations) on these mussels [23]. Proteogenomics data combined with bioinformatics tools provided key elements of biological context, such as the exact molecular and cellular processes triggered by carbamazepine exposure, where these molecular events were localized (e.g., organelle, cytoplasm, membrane, and extracellular), while helping us understand the observed changes at a metabolites level. For example, our metabolomics analysis revealed changes in fatty acid abundances, whereas the proteogenomics results highlighted up-modulation of the peroxisomal enzymes responsible for fatty acid β-oxidation [23]. In addition to providing an explanation for lipid metabolism disruption, these results also suggested the possibility of peroxisome proliferation leading to adverse effects on cellular homeostasis. Metabolomics data alone would not have been able to provide any further explanations.

The integrative view of molecular and cellular processes provided by multi-omics is also very helpful for PhAC MeOA determination. For instance, our previous findings led to a hypothesis on carbamazepine MeOA through autophagy induction in *M. galloprovincialis* (as in humans) via inositol depletion or endoplasmic reticulum stress [23]. Similarly, Ussery et al., applied a multi-omics approach (metabolomics and proteomics) and thereby obtained further evidence on the impact of the MeOA of guanylurea (metformin degradation product) on the early life stages of Japanese medaka [32]. After 28-day exposure at 1 ng/L concentration, they suggested that guanylurea acts by a MeOA similar to that of metformin, i.e., by activation of the AMP protein kinase (AMPK), as supported by the modulation of acetyl-CoA carboxylase 2 and alteration of the fatty acid abundance (e.g., stearic and pantothenic acid) downstream of AMPK.

Such mechanistic information could be useful regarding the AOP concept [15]. Indeed, metabolomics as well as other omics could facilitate the identification of molecular initiating events and early key events triggered by contaminant exposure that may lead to adverse outcomes at the individual level [40]. Omics can help delineate AOPs and therefore be a well-suited tool for hazard assessment. While the application of environmental metabolomics to investigate molecular effects and MeOA is highly promising for hazard assessment, many questions remain that PhACs (whose human MeOAs are known) could help identify. Indeed, findings on contaminant MeOAs in aquatic organisms require further consideration in the design of future experiments. For example, for a given PhAC, its MeOA could depend mainly on its concentration, exposure time, and the studied organism, as well as its sex and development stage.

The choice of concentrations likely to be present in the environment (ng-µg/L) would be preferred, as the toxicokinetics and toxicodynamics, and therefore the MeOA of a contaminant, may not be the same at a low or high concentration [41]. With regard to the choice of exposure time, MeOA could be schematized as a contaminant-induced sequence of molecular and cellular events that take place in a spatial and temporal framework, and lead to an adverse outcome at an individual level [15]. Hence, the choice of a short exposure time (in line with toxicokinetics) might be of interest for studying molecular initiating events or the early key events triggered by the contaminant. Otherwise, the choice of (semi-)long-term exposure could generate mechanistic information on more advanced key events close to the adverse outcome. Long-term exposure is also preferred when the study aims to correlate the metabolomics data with other measured biological endpoints at the individual level. However, these considerations have to be carefully taken into account, as the MeOA process is not always linear. Indeed, prolonged exposure could trigger other MeOAs over time, or a contaminant might have several MeOAs [15]. Finally, regarding the organism under study, it is essential to inform the development stages (e.g., adult, larvae, and embryo) [42], as well as the sex [21,26,35,43], as the physiological and morphological differences could impact the MeOA (e.g., endocrine disruptors).

These considerations regarding the design of experiments tailored for MeOA studies must be taken into consideration in order to coordinate efforts and generate results to complement existing knowledge.

## 6. Linking Metabolomics Data to Adverse Outcomes

Environmental metabolomics, especially when combined with other omics, may be effective for identifying MeOAs that have a high potential to contribute to the AOP concept and then to a more mechanistic-based hazard assessment of contaminants. To achieve this aim, it is essential for researchers to focus on determining the physiological roles of metabolites and how changes in their abundances are involved in different phenotypic outcomes [44]. Combining metabolomics with other physiological or morphological measures can be an effective way to confirm molecular mechanisms and start inferring the biological causality [44]. Interestingly, some studies have combined the metabolomics approach with other physiological or morphological measures to link metabolic changes induced by PhAC exposure to adverse outcomes at higher biological organization levels (Table S1 [20,21,25,26,29,33]). However, metabolomics is highly sensitive to subtle metabolic changes that may occur before any physiological, histological, or morphological modifications. Consequently, most of the studies reported metabolome changes in response to PhAC exposure, while no effects on the other measured endpoints (e.g., growth, weight, organ index, reproductive endpoints, etc.) were observed [20,29,33,34]. The exposure time therefore has to be long enough to be able to observe such physiological or morphological modifications. To date, only a few studies have succeeded in correlating metabolomics data with other measured biological endpoints in response to PhACs [21,25,26]. Among them, Davis et al., performed parallel analyses of reproductive endpoint measurements (i.e., plasma 17β-estradiol, testosterone, vitellogenin (VTG), VTG mRNA, and fecundity) and endogenous metabolites in the livers of fathead minnows (*Pimephales promelas*) exposed for 21 days to 5 and 50 µg/L spironolactone concentrations [21]. Based on a partial least square regression, in female minnows, several metabolites (i.e., L-carnitine and glutamate) were significantly correlated with fecundity and other endpoints that have been used as biomarkers of exposure to a variety of endocrine disrupting compounds (i.e., plasma VTG levels). These results were relevant, as glutamate is an important constituent of VTG in fathead minnows. Hence, the significant relationship between hepatic glutamate and fecundity was likely related to concomitant changes in plasma VTG [21]. Another study demonstrated behavioral changes in accordance with metabolite modulations in the gills of *Lampsilis fasciola* mussels exposed for 4 and 12 days to 0.005 and 1 µg/L 17α-ethinylestradiol concentrations [26]. The combination of metabolomics and behavioral approaches revealed upstream processes, such as changes in amino acid and nucleotide/nucleoside neurotransmitters, which might explain the reduction in female lure display behavior. Females that are unable to properly attract a fish host due to a lack of full lure display may not successfully contribute to reproductive success [26]. In a case involving a 7-day exposure of *Saccostrea glomerata* oysters to an estrogenic mixture (estrone, 17β-estradiol, estriol, 17α-ethinylestradiol, bisphenol A, 4-tert-octylphenol, and 4-nonylphenol), amino acid and carbohydrate metabolites were highly affected [25]. Such changes have consequent impacts on the citric acid cycle and lead to decreases in energy production (ATP). The authors noted a loss of body mass in oysters after exposure, in conjunction with the energy metabolism alteration. The authors thus put forward three possible hypotheses to be tested in further investigations on the ability of the MeOA of this estrogenic mixture to induce body mass loss: (i) estrogens may reduce biomass via estrogen receptor-mediated processes, (ii) estrogens may affect transcription of enzymes involved in the citric acid cycle, and (iii) estrogens may induce lower feeding rates resulting in fewer substrates available for the citric acid cycle.

The findings of these studies illustrated the ability of environmental metabolomics to generate meaningful information at a molecular level, which in turn can be linked to measurable physiological and morphological endpoints. The benefits of such an approach could therefore be tapped to gain insight into the molecular events triggered by contaminant exposure that lead to adverse outcomes at the individual level, as defined in the AOP concept [15].

## 7. MeOA Biomarkers to Enhance Environmental Biomonitoring

Metabolomics can also be applied as a diagnostic strategy to identify metabolic signatures (or biomarkers) specific to a given stressor or physiological or pathological state, which could be used in predictive models [45]. Such biomarkers may be suitable aquatic biomonitoring tools for generating early warning signals of environmental disturbances [8]. In environmental metabolomics, several authors focused on the identification of (potential) biomarkers in response to PhAC exposure. Our literature review revealed, however, that the main molecular effects highlighted in response to PhAC exposure and proposed as potential biomarkers (e.g., alterations in amino acid, nucleotide, and lipid and energy metabolism) were related to similar pathways that can be disturbed by other environmental contaminants (e.g., pesticides, polybrominated diphenyl ethers (PBDEs), polycyclic aromatic hydrocarbons (PAHs), polychlorinated biphenyls (PCBs), microplastics, heavy metals, and nanomaterials) [6,46,47] or environmental conditions [48,49]. It therefore may not be of interest to search for PhAC-specific biomarkers. A change of paradigm is needed as relevant molecular modifications are closely related to the contaminant MeOA. Indeed, MeOA-specific biomarkers should be identified (i.e., those that can be shared between several contaminants), instead of screening for biomarkers specific to a family of contaminants (e.g., PhAC and pesticides, which do not share the same MeOA within their own family). MeOA-specific biomarkers have been recommended for development in non-target aquatic species that are chronically exposed to PhACs [50,51]. By focusing more on the investigation of the key molecular events triggered by contaminants in line with their MeOA, environmental metabolomics (and multi-omics) could be more efficient for identifying a set of metabolites (and transcripts or proteins) as candidates for biomarkers specific to a group of contaminants sharing the same MeOA. In addition, it would be of interest to conduct research on MeOA-specific biomarkers in species representative of the biodiversity within various taxonomic groups (e.g., fish, molluscs, crustaceans, and amphibians), as such biomonitoring tools could then be directly applied under environmental conditions. Research on non-model species has been facilitated by advances in molecular techniques such as proteogenomics, which can overcome boundaries between model and non-model species [52].

Although only a few studies to date have generated such mechanistic information, it would be interesting to conduct a meta-analysis of metabolomics-based studies focused on contaminants with a similar MeOA (when identified), so as to extract a specific set of metabolites that could potentially be used as MeOA-specific biomarkers for environmental biomonitoring. Finally, before being proposed as a biomarker, a MeOA-specific metabolic signature has to fulfil several criteria (e.g., sensitivity, temporally sustainable response or known time-dependent response, distinguishable from confounding factors and biological variability, and reflection of a physiological state) [53], which would require further validation beyond metabolomics studies.

## 8. Recommendations and Prospects for Future Research

**Figure 2:**
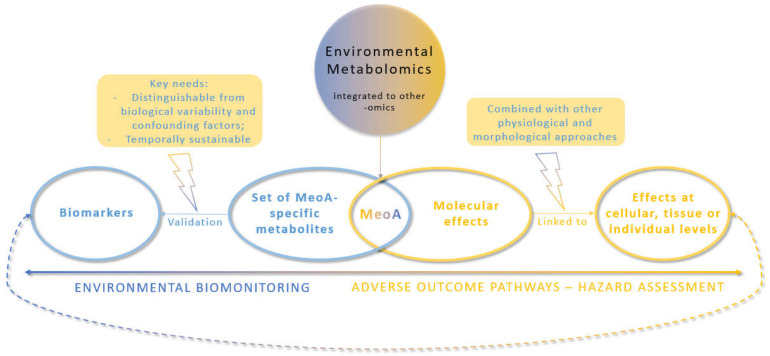
Environmental metabolomics strategy to generate meaningful and useful knowledge for hazard assessment and biomonitoring.

Despite the growing interest in environmental metabolomics to investigate the effects of contaminants on non-target organisms, we found that only a few studies provided all analytical, statistical, and biological information required to fully understand and interpret the metabolomics data. In order to overcome this issue, we thus recommend that authors list all information regarding the most statistically relevant features in Table S1. Essential information for mass spectrometry-based metabolomics experiments would be the (i) exact mass (mass to charge ratio; *m*/*z*), (ii) retention time, (iii) molecular ion species detected, and (iv) relative standard deviation calculated from the quality control sample, as well as, for each feature comparison, (v) the fold-change and (vi) *p*-value. Moreover, when features are annotated or identified, it is also necessary to provide the (vii) metabolite name, (viii) molecular formulae, (ix) mass default (in ppm) between the measured mass and the calculated mass, and (x) the level of confidence in the identification according to the criteria of Sumner et al. [17] or Schymanski et al. [54]. Finally, as metabolites should be placed in their biological context, Table S1 should also provide (xi) their associated metabolic pathways (or compound class when no metabolic pathway is available). Our review of the literature on PhAC also highlighted that many articles did not provide any information on the known MeOA of the studied compounds, and the authors did not offer any explanations with regard its potential MeOA in relation to their results. We strongly recommend doing this in order to provide a basis for further studies aimed at confirming (or not) the MeOA of a given PhAC under given conditions. In order to turn environmental metabolomics promises into achievements, we also recommend that future studies be carried out to further develop multi-omics integration as a key strategy for elucidating the MeOA of PhACs (and contaminants in general), and to help link molecular initiating events to adverse outcomes at higher biological organization levels. Good knowledge of the MeOA would benefit hazard assessment to fill AOPs and enhance environmental biomonitoring by implementing MeOA-specific biomarkers (Figure 2). Such biomarkers could generate information on the MeOA of contaminants present in the environment and on the physiological state of organisms or the likely adverse effects. These recommendations concern all environmental contaminants, not only PhACs.

## Supplementary Materials

The following supporting information can be downloaded at https://www.mdpi.com/article/10.3390/metabo12020186/s1. Table S1: Overview of the environmental metabolomics results (and metadata) related to the exposure of aquatic organisms to pharmaceutical active compounds (PhACs), as well as the interpretation of results.
